# Spirituality in Patients at the End of Life—Is It Necessary? A Qualitative Approach to the Protagonists

**DOI:** 10.3390/ijerph19010227

**Published:** 2021-12-26

**Authors:** E. Begoña García-Navarro, Alicia Medina-Ortega, Sonia García Navarro

**Affiliations:** 1 Nursing Department, University of Huelva, 21007 Huelva, Spain; aliciadelosmaresaxo@gmail.com (A.M.-O.); soniagarcianavarro@gmail.com (S.G.N.); 2 Social Studies and Social Intervention Research Center (ESEIS), Contemporary Thinking and Innovation for Social Development Research Center (COIDESO), Faculty of Nursing, University of Huelva, 21007 Huelva, Spain; 3 Coping In the End of Life Reseach Center (AFLV), INVESTIGA+, Junta de Andalucía, 41008 Sevilla, Spain; 4 United Clinical Management (U.G.C. Los Rosales), Distrito Huelva-Costa-Condado-Campiña, 21007 Huelva, Spain

**Keywords:** spirituality, palliative care, nursing skills, end-of-life

## Abstract

Spirituality is the most unknown aspect of palliative care despite being the need that is most altered in the last moments of life. Objective. To identify on the one hand the spiritual needs of patients who are at the end of life and on the other hand, the way in which nursing professionals can work to provide effective accompaniment in this process. Method. A qualitative study was conducted which applied different data collection techniques. This was done to describe the phenomenon from a holistic perspective in relation to experts’ perceptions of the competencies required by health professionals and palliative patients’ spiritual needs. Semi-structured interviews were conducted within both populations. In order to analyze the qualitative data collected through interviews, discourse was analyzed according to the Taylor–Bodgan model and processed using Atlas.ti software. Results. Three well-differentiated lines of argument are extracted from the discourse in each of the groups, on the one hand in the group of patients they define the concept of spirituality, system of values and beliefs, and the Factors that influence the spirituality of patients at the end of life (differentiating palliative care areas/other areas) and on the other, the professionals agree with the patients in the line of argument of concept of spirituality although they define more metaphysical categories and the other two lines of argument that result are the spiritual attention in this process and the need for formation in spirituality. Conclusions. The provision of spiritual care gives meaning to the actions of nursing professionals when it comes to providing end-of-life care, achieving holistic care, humanizing death, and promoting a dignified end.

## 1. Introduction

In a first approach to the word Spirituality, it is necessary to mention that it comes from the Latin spirit, which means breathing, vitality. If this concept is related to the word Alma, in Latin anima, it means the capacity for transcendence [1]. A more contemporary definition is described by Mytko and Knight [2] as a set of feelings that lead the individual to connect with himself, with others, with the purpose of life or with nature in search of value and meaning, to find peace and harmony. These authors mention the difference between the constructs of Spirituality and Religiosity, indicating that they are not exclusive of each other, that they can overlap or exist separately, as long as, carefully, they are categorized and interpreted. Under this same conceptual line, Puchalski describes spirituality as the aspect of the human condition that refers to the way in which individuals seek and express meaning and purpose, as well as the way in which they express a state of connection with the moment, with oneself (self), with others, with nature and with the significant or Sacred [3].

The appreciation of the spiritual dimension and the inclusion of professionals trained in palliative and spiritual care capable of listening to fears and pain providing hope and recognition to terminally ill patients are the basis of spiritual care models. This model of care has its foundations in the biopsychosocial–spiritual model of care [4] and the patient-centered model of care [5].

Spirituality is the least understood aspect of end-of-life care despite, surprisingly, being the most altered need in this process. Spirituality is a priority in the fundamental objectives of palliative care work. Palliative care focuses on improving the patient’s quality of life, and this cannot be favored as a whole if the spiritual dimension is not addressed [6]. In the words of Stanislav Grof: *“Spiritual development is an evolutionary capacity that is innate to all human beings”*. It describes an impulse towards totality and the discovery of one’s true potential [7]. It is as common and natural as birth, physical growth, and death, being an integral part of our existence. It is a classic concept, in the same way as the concept of human beings and their fear of facing death and the search for vital meaning. This means that, at the end-of-life, this fear and anguish grows exponentially making spiritual care hugely important for terminally ill patients [8]. For patients in palliative circumstances, spirituality is considered as a driving force to provide an optimal response to the circumstances of these individuals in relation to their own existence. “The practice of spirituality is also seen as an agent for the transformation and regulation of emotions, with this representing an effective tool for reducing levels of depression and anxiety in those who find themselves in the final stages of life” [9].

A high percentage of hospitalized patients are faced with emotions such as anguish, fear, depression, anger, dissatisfaction, etc., which are emotions with a high emotional and spiritual load. Nursing must care for these emotions [6]. According to studies conducted by Silvia Caldeira, *“spiritual anguish is defined as a state of suffering which is related with a lack of meaning in life*” [10]. In order to tackle this diagnostic, spiritual care must be provided by nursing professionals. This care should consider the way in which professionals are and act with both patients and their families. It should, therefore, be perceived as a holistic dimension of palliative care which preserves dignity and facilitates patients in their search for vital meaning and the relief of suffering [8].

It should be recognized that this sphere is a competence which must come, not only from psychology professionals belonging to the palliative care team but, also, from within the entire multidisciplinary team [11]. Different studies show that the nursing plays an important role at the time of facing death. Facing death appropriately poses the need to know the best way to act when faced with situations which generate great suffering and anxiety. This is the case as much for the individual living the end-of-life process, as for the professionals accompanying them and conditions their approach to providing care fitted to patient needs. An appropriate approach helps patients have a good death [10,11,12].

At its heart, all nursing care provision is based on spirituality as it is guided by hope, compassion and the conviction that an individual’s life remains full of possibilities, even though it is limited in certain aspects [12,13]. Nonetheless, some challenges are encountered in real life. Indeed, despite spiritual care being an integral part of nursing care, it’s provision is highly diverse and can be influenced by the individual, cultural, and educational background of each nurse [13]. In the same way, a number of prejudices with regards to nursing are found in the healthcare system. Nurses have been judged to have underestimated the spiritual dimension in care and various factors have been proposed to explain this limitation. These factors include a lack of awareness of its importance and a lack of preparation, incorrect interpretation of the term spirituality, lack of desire to provide spiritual care. However, different systematic reviews have served to demonstrate [8,9,10,11,12,13,14] that in the nursing setting, nurses demonstrate an understanding of spiritual and religious care that is in tune with the construct that is advocated in the present day as much in the current Spanish setting as in Europe and the United States. Professionals have also indicated that patients serve as a mirror of their own mortality and, when health workers are open to listening and sharing anguish, they are able to understand the process gone through by patients and facilitate the search for making sense of the illness itself. Nevertheless, professionals who are not able to face up to their own problems with regards to death will find it even more difficult to face up to the death of another person and with seek to distance themselves from it [15]. The attitudes of nurses towards this type of care is favorable, although a need to raise awareness of specific related care responsibilities is detected. Training relating to some aspects of intervention needs to be completed [16].

With the development of the study we propose on the one hand to identify the spiritual needs of patients in a situation of terminality through their discourse and on the other, by the hand of experts in palliative care, to know what professional skills are decisive to respond to the needs of patients at the end of life. In this way, the foundations would be laid for future lines of research aimed at establishing proposals for improvement based on evidence.

## 2. Materials and Methods

A qualitative design and phenomenological approach was followed through content analysis as described by Taylor and Bodgan [17]. We have used this methodology since it allows us to know the emic of the participants, that is, to understand the perception of people, as well as feelings and thoughts, from their testimonies, full of meanings, symbols, intentions, motives, and beliefs. Esta investigación se adhiere a las directrices del COREQ [18].

In order to respond to the proposed objectives, we address two populations. On the one hand, we selected palliative patients belonging to care support groups in the municipality and on the other hand we selected expert professionals that worked in in end-of-life processes in the same geographical setting. The sampling strategy was theoretical sampling, a technique that was developed by Glaser and Strauss [19] and where the sample is selected through the use of a successive strategy, progressive incorporation of informants, and evidence of similar studies, coinciding in a total of 10 expert professionals. However, in the group of patients, given the difficulties of accessibility to palliative patients voluntarily reported to be informants, the snowball sampling technique was used [20], we were able to reach 7 patients who behaved in a heterogeneous sample, and given their generosity and need for expression we managed to saturate in each of the dimensions found.

Intentional sampling was carried out during the months from May to November 2020. The sample size was determined progressively during the course of the research incorporating informants until the saturation of the information was reached [21]. In other words, research activities were continued until no new data pertaining to thematic categories could be extracted. In order to be considered for inclusion, this patient group had to comply with the criteria of belonging to the municipality under study, being of adult age, having been informed of their diagnosis and prognosis, having been informed about the process to be undergone, and, finally, having signed an informed consent form. In the case of expert professionals in end-of-life processes, once relevant individuals agreed to participate, the only criterion was that they provided written informed consent.

In order to analyze the discourse obtained in the interviews, the model described by Taylor–Bodgan [17] (Table 1).

The research team meets to obtain the script of the interviews and we carried out a pilot test with a professor and a collaborating student. Subsequently, the principal investigator conducted in-depth interviews with the aim of further exploring some of the dimensions that had emerged in the first interviews and thus obtaining more data.

Interviews were recorded and transcribed. The research team listened to and read the interviews in order to make an initial superficial interpretation. This provided a general idea which supported a more in-depth analysis (identification of relevant recurring themes, search for similarities and differences between themes in order to develop codes-dimensions and, with these, thematic categories. The repetition of codes—dimensions—on behalf of researchers—blind analysis—indicated that the analysis got to the essence and exposed the meaning of the studied phenomenon).

The two study populations were made up of patients and expert professionals in end-of-life processes. In order to ensure validity and reliability, the entire process of coding and analyzing discourse was conducted independently by three members of the research team. Discrepancies were discussed until consensus was reached.

The study was conducted according to the guidelines of the Declaration of Helsinki, and approved by the Ethics Committee PPEIBA, government of Andalusia, Spain (protocol code 01/2020 CEPP and date of approval 18 January 2020).

## 3. Results

### 3.1. Participant Characteristics

Seventeen informants were interviewed. This included seven patients, of both sexes and with different illness progression states, undergoing palliative processes. This also included ten end-of-life expert professionals, these were representative in terms of age, gender, and years of experience in the field (Table 2).

The coding of discourse from interview transcripts produced 27 different codes. Of these, 17 were generated in the patient group and 10 in the expert population. This basis was developed using the data management program Atlas.ti. In consideration of the repetition of utterances in relation to each code, six lines of argument emerged which were equally distributed within both population groups, suggesting that the concept of spiritually coincided in each population (Table 3).

### 3.2. Lines of Argument

Line of argument 1 (patient group), coincides with line of argument 4 (professional expert group) with regards to the concept of spirituality. This agreement is not so much seen in the content overall, which is understandable given existing differences between both populations, but in reference to the search for the meaning of life as a cornerstone of spirituality (Table 4).

Considering further the idea of individual projection used by the palliative patient group to discuss spirituality relative to end-of-life processes, a code emerges which reached saturation very quickly. This code referred to connecting with others in order to give meaning to the processes. Selected patients also added accompaniment and communication to this concept.

*“I am really well attended to by all of my family. My sister-in-law comes every other day to see me and asks me many questions. I am grateful to my wife for all of the help and love that she is giving me, and of course for caring for me completely. I feel really loved by the people who are around me, they really go out of their way”.*

*(Patient Palliative 02)*

*“I tell my daughter not to suffer because of me, that she has to live he r life. My daughter gets scared a lot because she thinks that I am going to bleed out and I have to cheer her up”.*

*(Patient Palliative 04)*

Turning attention back to the patient group, a second line of argument is found which was called as system of values and beliefs, and was deemed to be fundamental for understanding the spiritual end-of-life component. As with the other arguments generated by this population, the intensity of this component meant that it was tightly linked with the moment being lived at the time of data collection. Further, this line of argument defined codes that were highly relevant to end-of-life accompaniment. On the one hand, the system of values and beliefs emerged at a general level. On the other hand, something more metaphysical was coded. It is important to highlight that all of the discourse transcripts were coded as highlighting this component, with saturation occurring prematurely following discussion of faith and religion.

*“I believe so, the idea that there is someone and that I turn to them at certain times. I believe in a higher being, accompaniment exists at certain moments. Yes at times when I need them, when it gives me stability. But I am not practicing at all”.*

*(Patient Palliative 02)*

Another dimension, linked with one’s belief system and, at the same time, spirituality, that was generated by informants was hope.

*“I when I lead a normal and acceptable life I feel super satisfied, I have learned to enjoy the small things. Get up and do three things in my house, it’s the most wonderful thing in the world to me, because maybe in the future I won’t be able to. I have hope again. I am able to cling onto anything. With really small things, share time with others. I think that that is really positive and it helps me a lot in these moments”.*

*(Patient Palliative 01)*

The palliative patient population also coincided with the expert professional population with regards to the need for palliative care to include attention to spirituality. Differences emerged in patient discourse which pertained to the approach taken in palliative units and in other units.

*“In palliative patient they do it really well, because they approach that facet, they treat your symptoms, but also your concerns”.*

*(Patient palliative 03)*

The last line of argument generated from patient discourse, Line of argument 3, pertains to the factors that influence patient spirituality at the end of life. This argument is characterized by a large volume of emergent dimensions, with all being born out of service user perceptions. Saturation was reached with regards to the need to address conflict (Table 5).

With regards to the expert professional group, we previously described a line of argument that is in sync with another given by patients; however, another two arguments stand out for revealing idiosyncrasies of this group. Namely, these are spiritual care and training needs for effectively providing this care.

One of the subcategories underlying this first argument is spirituality in clinical and spiritual care. Utterances gathered together in relation to this dimension came from discourse that focused on a need that is currently not catered for, not even in palliative care.

*“I’ll sum it up in a really simple way, if when the moment comes you are not capable of standing 25 minutes of hugs and grief with a person in your arms, you’re not qualified. We are used to working with the pain ladder and when one approaches on an existential level the reality of a person who is dying they must be able to open up, to tremble with the other and above all to look with them into the abyss, I think that it isn’t done well because I think that we live with little awareness us professionals”.*

*(Professional expert 08)*

*“Because there isn’t the custom of talking about this, and less in those moments at the end of life. For me it was much more difficult to do it at other services where there was a lot of need because it wasn’t expected of me, in the palliative context it is. But there isn’t any assessment of the life project, religious dimension, celebrations, last rites; rites are really important to the spiritual dimension. It isn’t integrated as a part of the job. Because there is a need to respond to what is expected of the nurse”*

*(Professional expert 02)*

Other experts, in this same category, had already pointed to training as the answer to integrating the spiritual component into end-of-life care. The absence of this care is linked with the scarcity of competencies in this area.

*“If it isn’t taught or if no experience is given of spirituality in the teaching of medicine or nursing, well we run the big risk of putting ourselves in front of a sick person without criteria that help us to understand beyond what is presented in their illness. It is never our job to judge anybody, but to be the doctor to whom the person can flow what they really want to express from inside of them. And all of this in a climate of confidence and serenity. Embodied spirituality is also this expression of support in daily things that make life more dignified”.*

*(Professional expert 10)*

Finally, the presentation of results is ended with the sixth line of argument, which was alluded to in all of the aforementioned categories and for which a code emerged following specialization of discourse. This code refers to the training needs of professionals to approach the spirituality of patients who find themselves undergoing end-of-life processes. Some utterances agreed on the need to know oneself and undergo introspection in order to be able to attend to another.

*“The patients open up the path to this search and I study. A lot of things come out of your own free will, but, are you doing it right or not? It isn’t easy to bring serenity to someone who is dying, that this process that they go through is a process of personal and family growth, if you yourself don’t believe it. First you have to wake up within yourself, then you have to train yourself to not do things wrong, because without wanting to you can do harm”.*

*(Professional expert 04)*

Other utterances refer to the need to conduct team training in an interdisciplinary way in order to be able to provide holistic patient care.

*“From our daily experience in hospitals at no time is a formal meeting space found where it is possible to work on teamwork in all of the required areas. And this is a mistake because it limits a lot the reach of care to patients and to those around them. Medical care and nursing slip into healthcare, but the spiritual is not integrated”.*

*(Professional expert 07)*

## 4. Discussion

The two populations under study in this research coincide in defining spirituality, in describing it as a way to connect with others and give meaning to the final process they are experiencing—in the case of palliative patients—or to give meaning to their existence and contact with life in the case of expert professionals. Authors, like Torralba [22], define spiritual intelligence as a form of existential or transcendent intelligence which enables human beings to question the meaning of their existence. It allows them to step away from reality, favors the elaboration of a life project, and permits them to transcend materiality, interpret symbols and understand the wisdom of life.

Another of the dimensions described in the results where both groups, coincide is the need to addressing the spiritual component in end-of-life care. Various authors [3,4,6] agree that treatment that strives to provide integral and dignified care must attend to all dimensions of the human being and tackle all patient and family needs. It should converge to include “dying well”, the absence of suffering (in terms of the physical, emotional and spiritual) and the absence of pain for both the inflicted individual and their family. The present study, in accordance with a previous study [3], verified that these spiritual needs are present during the final moments, even when the patient is not aware of it. This is exemplified by the comment: “*I have learned to enjoy the small things. Get up and do three things in my house, it’s the most marvelous thing in the world for me, because maybe in the future I won’t be able to*” (Patient Palliative 01). Other previous research studies have also revealed similar outcomes [11,12]. Torralba [22,23] named needs of a spiritual nature that were conceived by the philosopher Simone Weils [24], in which the need to find meaning during these moments of life and reconciliation with oneself require spiritual intelligence. These can also be extrapolated from the discourse provided by unwell individuals in response to the situation in which they find themselves as a means to overcome it.

Delving deeper into the narrative of interviewed patients undergoing end-of-life processes, two main worries or concerns emerged with arise at these moments [22]. Specifically, these were concern around what death would be like or what would take place at the moment of death (Patient palliative 01) and fear of suffering (Patient Palliative 04). At the same time, references to spirituality emerged in all aspects of life as a method of help and support. This is demonstrated in the quote: “*A lot of pain, but always positive, wanting to better myself, wanting to be strong and smiling for my husband, for my son, for my parents and my sister*…” (Patient Palliative 03). Similar outcomes have also been described in works conducted in the clinical setting [3,25].

Based on the discourse resulting from the content analysis of expert professionals, the physical and emotional vulnerability developed to end-of-life processes causes an increase in the spiritual needs of patients and their families. These needs must be satisfied by health professionals in the end-of-life care setting. These results coincide with previous studies [3,4], that spiritual needs permeate the discourse of informants along with the importance of addressing them in order to provide comprehensive and exemplary care to patients and families involved in palliative care.

Besides, outcomes of the present study demonstrate the importance of spirituality at these moments and the huge impact of the quality of care, in the same way as reflected in other research [3]. It can be concluded that, based on the discourse of participating informants, the spiritual dimension is a felt need pertaining to the population of palliative patients in Huelva.

According to the palliative patients interviewed, nursing professionals must attend to the spiritual needs of patients at the end of life. There are authors [26] who agree on this idea, and conclude the need to include it in the academic world. An idea that in turn coincides with the speeches provided by the expert professionals interviewed. For instance, “*I have the feeling that this dimension is not touched upon not even when covering other material. The spiritual dimension is not integrated in the curriculum anywhere, therefore, nobody has to give it*” (Professional expert 02). The study confirms that spirituality is forgotten about. Nonetheless, it is true that students present high indices of knowledge and positive attitudes towards spirituality [8], although deficiencies are observed in knowledge and the delivery of nursing interventions related with spirituality [11]. New generations of students will be the future of the nursing profession. If these students are not trained to meet this need, spirituality will once again become an empty space in “integral” care plans.

With regards to discourse provided by professionals, it was revealed that positive attitudes exist along with a predisposition towards spiritual care. Such spiritual care is not only for religious individuals but also includes atheists and agnostics. This is in accordance with the concept of holistic health care proposed by the World Health Organization, which includes the integration of spirituality in nurses’ care plans in line with previously conducted studies [12].

According to various studies [3,11] and two of the interviewed experts (Professionals Experts 01, 05), the act itself of working at a palliative care unit, where professionals continuously come into contact with death, should help them develop skills to provide spiritual care. In this sense, the compassion satisfaction shown by the care provider to the sick person connects with that individual and, at the same time, helps the professional manage their own feelings and compassion satisfaction [27].

The concept of spirituality defined by the panel overall agrees with current understanding of the construct, not only in the Spanish context but, also in Europe [7,13,22,28]. “Spirituality, which does not identify with a single unique divinity, is the expression of the essence of a person from where everything is governed and finds value” (EXP05). Spiritual care is understood as being basic to nursing care, as indicated by: “Each one is how they are, this generates suffering at the time of death and if you as a professional don’t mitigate that, death is not as dignified as it should be” (Professional expert 01); “Spirituality is the capacity to tremble with the other person” (Professional expert 04). The importance of training in this environment was emphasized, in both nursing degrees and postgraduate training, and of the use of therapeutic tools and tools to detect spiritual suffering. This was outlined in the quote: “But we realized that it wasn’t just an exploratory tool but a therapeutic tool. Those who use it must be really aware, be in control, know how far to go, when they have to interrupt, when is the right moment and when no, for this you have to be trained” (Professional expert 03). Similar outcomes have been reflected in various articles [3,4,6,24,29].

### Strengths and Limitations

This study is innovative as it describes the need for spiritual accompaniment in the final process of life from the perspective not only of the professional who performs it, but of the protagonists themselves, the patients who are in this process. More studies and research, both quantitative and qualitative, are needed that contemplate the spiritual need, as well as the competences of nursing professionals for their development both in our field of study—palliative care—and outside it. Therefore, this research should be extended to different health professionals, doctors, nurses and psychologists, as well as to different areas where addressing the end of life is a priority.

Social awareness, and specifically of health professionals, in the spiritual field is fundamental, so this content should be included in the most initial stages of individual education. “A society without spirituality is dead, breathless, without criteria, it allows itself to be manipulated by whatever ideology it comes from. That is why a mature spirituality makes the person and society critical, open, non-manipulable, constructive: free”.

## 5. Conclusions

The provision of spiritual care gives meaning to the actions performed by nursing professionals and the end of life, achieving holistic care, humanizing death, and promoting a dignified end. In the present work, it is true that spiritual accompaniment was seen to be challenged by its very nature given that the experimental paradigm did not achieve complete understanding or exploration. This being said, obtained outcomes verify that the spiritual dimension is understood by professionals on hand to accompany as a human universal and, with that, approaching it correctly will help other needs to be addressed. For this reason, following elaboration of the present study, it can be concluded that better training is required in this setting. Such training should be transversal, and be included within the Nursing degree, as well as in postgraduate training. This would promote the lifelong learning of nursing professionals in the city. In this way, social awareness could be strengthened within the nursing context in a way that encourages professionals working in the field to contemplate spiritual accompaniment as an indispensable aspect of care plans.

In the run up to the end of life, a lack of spiritual care becomes even more tangible for patients. Through the present study it can be confirmed that not only palliative patients are impacted by this dimension and, instead, spiritual conflict take occur at any vital stage and generate suffering. Nursing must be on hand to meet this need and mitigate its potential consequences through the route of integral and personalized care.

## Figures and Tables

**Table 1:** In-process analysis approach in qualitative research (Talor–Bogdan).

Stage	Action
Discovery (search for topics by browsing the data in every possible way)	Read the data repeatedly Keep track of themes, insights, interpretations and ideas Look for emerging topics Build topologies Develop concepts and theoretical propositions Read the bibliographic material Develop a history guide
Coding (meeting and analysis of all the data that refer to themes, ideas, concepts, interpretations and propositions)	Develop coding categories Encode all data Separate the data belonging to the various coding categories See what data has been left Refine your analysis
Data revitalization (interpret them in the context in which/where they were collected)	Requested o unsolicited data Observer influence on stage Who was there? (differences between what people say and do when are alone and when there are others in the place) Direct and indirect data Source (distinguishing between the perspective of a single person and that of a larger group Our own assumption (critical self reflection)

**Table 2:** Sociodemographic characteristics of those interviewed.

Participant	Patient/Professional	Age	Disease Evolution Palliative Care Experience (Years)	Sex
Participant 1	Palliative Patient	74	4	Female
Participant 2	Palliative Patient	78	2	Male
Participant 3	Palliative Patient	45	0	Male
Participant 4	Palliative Patient	64	6	Female
Participant 5	Palliative Patient	69	3	Female
Participant 6	Palliative Patient	51	4	Female
Participant 7	Palliative Patient	60	3	Female
Participant 8	Nursing Professional	53	17	Female
Participant 9	Academic Professional	47	17	Female
Participant 10	Nursing Professional	60	28	Female
Participant 11	Nursing Professional	49	24	Female
Participant 12	Nursing Professional	56	28	Female
Participant 13	Nursing Professional	55	24	Male
Participant 14	Academic Professional	34	10	Female
Participant 15	Nursing Professional	41	16	Female
Participant 16	Nursing Professional	48	22	Female
Participant 17	Nursing Professional	56	26	Male

**Table 3:** Origin of study categories and subcategories according to population group.

Study Population	Line of Argument	Categories	Work Experience	Scientific Evidence	Emerging Discourse
Palliative care patient	Concept of spirituality	Meaning of life	*	*	
		Connection with others	*	*	
		Hope			*
	System of values and beliefs	Patient’s system of values and beliefs	*	*	
		System of values and beliefs linked with religion and metaphysical phenomenon			*
		Spirituality in end-of-life care	*	*	
		Spirituality in society			*
	Factors that influence the spirituality of patients at the end of life	Worries/concerns	*	*	
		Conflict			*
		Coping strategies			*
		Social support	*	*	
		Sense of security	*	*	
		Proximity to death	*		
		Feelings of despair			*
		Pain	*	*	
		Fear of suffering	*	*	
		Body image			*
		Family background			*
End-of-life processes: Professional	Concept of spirituality	Spirituality	*	*	
		Evolution of spirituality			*
	Spiritual care	Spirituality in clinical care	*	*	
		Spirituality in palliative care units			*
	Training needs pertaining to spirituality	Need for spirituality training	*	*	
		Degree training			*
		Postgraduate training			*
		Personal experience			*
		Tools to evaluate patients’ spiritual needs			*

The * symbol indicates that each category described has been generated in the different techniques shown in the column headings: Work Experience, Scientific Evidence y Emerging Discourse.

**Table 4:** Line of argument 1 patients: Concept of spirituality vs. line argument 4 Professional expert: Concept of spirituality.

**Line of Argument 1** **Vs.** **Line of Argument 4**	**Role**		
	Professional expert	EXP02	*“It is the need that men and women have to transcend daily life, giving it meaning. The same with passions, that can be related with the image of some type of God or specific ideas… that people can demonstrate solidarity with each other is nothing more than the most beautiful or wonderful branches of spirituality. It is a dimension that is within all human beings, and not only in each human being, but also in every town”*
		EXP07	*“Spirituality, which does not identify with a single unique divinity, is the expression of the essence of a person from where everything is governed and finds value. A person who does not have spirituality bears a higher burden of internal defeat. They lack the foundations and nutrients that help to interpret life… A society without spirituality is dead, it gets manipulated by whatever ideology regardless of where it comes from. For this, mature spirituality leads individuals and society to be critical”*
	Palliative Patients	PPAL01	*“I am very satisfied with the life I have led before the illness, and with the life I lead now. I go out, I don’t hide myself away at home. I don’t ask for things I can’t do. I don’t have that feeling of “I could have done that and I didn’t do it”*
		PPAL03	*“I know everything I have, I don’t stop asking because I want to know how long I have left to live and how I am going to be up until I die”*
		PPAL07	*“But I want to be awake up until the end if I don’t have strong pains. Until the end I want to see my children, my grandchildren, my neighbors, my daughters-in-law. I wouldn’t like to lose my mind or say silly things”*

**Table 5:** Line of argument 3, palliative patient group. Factors that influence patient spirituality at the end of life.

**Line** **Argument:** **Concept of Spirituality**	**Role**			
	Palliative Patients	need to address conflict	PPAL3	*“People are not really used to expressing anything that is not exactly physical, for this reason I try to resolve conflict both talking and without talking”*
		coping strategies	PPAL6	“*I am dealing with it 100% well, don’t consider it, I move forward, I keep going. I am great, I always say that I am good, it is better to not dwell*”
		social support	PPAL3	“*My husband is my main support, because my children support me, they call me every day, but they live in Madrid. They really do care for me a lot. But without him I wouldn’t have had the strength to go on“*
		sense security	PPAL7	“*It makes me feel good being able to lead the most normal life possible. To be able to have a beer someday with friends, any little thing will do for me*”
		closeness to death	PPAL2	*“I know I’m in a very advanced stage of metastasis but then... how it’s going to be, what I’m going to feel...... I think everything will be fine and that relaxes”*
		feelings of despair	PPAL4	*“But yesterday got me a bit despairing, and I said “now whatever has to happen”. Plus I really wanted to cry and I said “now I can’t stand this anymore”*
		pain	PPAL2	*My legs I can hardly move them for the pain. And for this I take a lot of painkillers every day, that has morphine, up until now they haven’t given me a single day without pain. They tell me that my pain is very difficult because it is in the bones and the nerves*”
		fear of suffering	PPAL1	“*Ay goodness me, that I don’t have to suffer much when I am dying, that my loved ones don’t see me suffer so that they don’t suffer, that I fall asleep one night, but the suffering*…”
		body image	PPAL7	“*7 years ago now I had an operation for breast cancer, they gave me a prosthetic and it looks awful, looking at it, it’s the difference between the two that you notice even with clothes, but it doesn’t bother me as much now because I have other concerns*”
		family background	PPAL5	“*Four siblings have died of the same thing. And my mum, I think it is hereditary. There’s more to come…I had a really bad time when they went, with my twin, we always used to go out together…. It is obvious that my destiny is what it is, the same as my family*”
